# Neurotranscriptomics: The Effects of Neonatal Stimulus Deprivation on the Rat Pineal Transcriptome

**DOI:** 10.1371/journal.pone.0137548

**Published:** 2015-09-14

**Authors:** Stephen W. Hartley, Steven L. Coon, Luis E. Savastano, James C. Mullikin, NISC Comparative Sequencing Program, Cong Fu, David C. Klein

**Affiliations:** 1 Comparative Genomics Analysis Unit, Cancer Genetics and Comparative Genomics Branch, National Human Genome Research Institute, National Institutes of Health, Bethesda, MD 20892, United States of America; 2 Section on Neuroendocrinology, Program in Developmental Endocrinology and Genetics, *Eunice Kennedy Shriver* National Institute of Child Health and Human Development, National Institutes of Health, Bethesda, MD 20892, United States of America; 3 Department of Neurosurgery, University of Michigan, Ann Arbor, MI 48109, United States of America; 4 National Institutes of Health Intramural Sequencing Center, National Human Genome Research Institute, National Institutes of Health, Rockville, MD 20852, United States of America; Morehouse School of Medicine, UNITED STATES

## Abstract

The term neurotranscriptomics is used here to describe genome-wide analysis of neural control of transcriptomes. In this report, next-generation RNA sequencing was using to analyze the effects of neonatal (5-days-of-age) surgical stimulus deprivation on the adult rat pineal transcriptome. In intact animals, more than 3000 coding genes were found to exhibit differential expression (adjusted-p < 0.001) on a night/day basis in the pineal gland (70% of these increased at night, 376 genes changed more than 4-fold in either direction). Of these, more than two thousand genes were not previously known to be differentially expressed on a night/day basis. The night/day changes in expression were almost completely eliminated by neonatal removal (SCGX) or decentralization (DCN) of the superior cervical ganglia (SCG), which innervate the pineal gland. Other than the loss of rhythmic variation, surgical stimulus deprivation had little impact on the abundance of most genes; of particular interest, expression levels of the melatonin-synthesis-related genes Tph1, Gch1, and Asmt displayed little change (less than 35%) following DCN or SCGX. However, strong and consistent changes were observed in the expression of a small number of genes including the gene encoding Serpina1, a secreted protease inhibitor that might influence extracellular architecture. Many of the genes that exhibited night/day differential expression in intact animals also exhibited similar changes following *in vitro* treatment with norepinephrine, a superior cervical ganglia transmitter, or with an analog of cyclic AMP, a norepinephrine second messenger in this tissue. These findings are of significance in that they establish that the pineal-defining transcriptome is established prior to the neonatal period. Further, this work expands our knowledge of the biological process under neural control in this tissue and underlines the value of RNA sequencing in revealing how neurotransmission influences cell biology.

## Introduction

The rodent pineal gland is an especially attractive model for neurotranscriptomic studies because of evidence of large neurally-regulated changes in the abundance of many pineal transcripts [1–9]. The gland is innervated by sympathetic projections from the superior cervical ganglia (SCG) [10–13]. At night norepinephrine is released in response to signals originating in the hypothalamic suprachiasmatic nuclei (SCN), which house an autonomous circadian clock. The SCN are hard wired to the pineal gland by a multisynaptic neural pathway: SCN neurons project to the paraventricular nucleus, where they contact neurons that project caudally via the mesencephalic periaqueductal gray to the intermediolateral nuclei in the upper thoracic segments of the spinal cord [14–16]. From there, preganglionic neurons contact a small subpopulation of SCG cells which innervate the pineal gland via projections through the internal carotid nerve and the conarian nerves. Light acts on this system through the retina and a retinohypothalamic projection that terminates in the SCN; light entrains the SCN clock to environmental lighting and also gates stimulatory signals to the pineal gland [14, 17–20]. Experimental deprivation of neural stimulation of the pineal gland can be accomplished by removal (SCGX) or decentralization (DCN) of the SCG, thereby eliminating circadian input from the SCN [21].

Norepinephrine is the primary biogenic amine controlling the pineal gland and acts through an α_1b_- and β_1_-adrenergic receptor “AND” gate mechanism to activate adenylate cyclase and elevate cyclic AMP [22–28]. Whereas β_1_-adrenergic receptor activation is essential for activation of adenylate cyclase, this effect is potentiated by α_1b_ –adrenergic receptors via Ca^++^ and phosphatidyl inositol (Pi) activation of protein kinase C. The adrenergic, receptor-dependent elevation of cyclic AMP activates protein kinase A, which in turn phosphorylates cyclic AMP response element binding protein, which is bound to regulatory elements, thereby triggering gene expression [27, 29–31]. In addition to releasing norepinephrine, adrenergic neurons serve to take up and sequester norepinephrine and other catecholamines; this prevents stimulation by circulating catecholamines entering the pineal perivascular space, thereby enhancing the on/off nature of neurotransmission [32]. Accordingly, whereas both SCGX and DCN block stimulation of the pineal gland, the reuptake/sequestering function of the sympathetic nerves is retained following DCN and is eliminated by SCGX [32]. In addition to neural regulation of the pineal transcriptome, peripheral clock mechanisms operating within the tissue may play a regulatory role.

Previous neurotranscriptomic studies of the pineal gland have used a variety of classical biochemical methods and cDNA microarray technologies [2, 5, 9, 33, 34]. In the current report, strand-specific next-generation RNA-Seq is used to extend this body of knowledge. This powerful technology provides unprecedented statistical power across a broad dynamic range, allowing robust detection of differential effects in more genes than ever before [35–38]. This effort has increased our knowledge of the rodent pineal gland by identifying genes not previously known to be under neural control. Examination of the effects of neonatal (5-days-of-age) SCGX and DCN on the adult rat pineal transcriptome revealed that these procedures essentially eliminate the 24-hour pattern of differential gene expression but do not markedly alter the developmental expression of the non-rhythmic component of the pineal-defining transcriptome.

Our results provide a powerful new resource for investigators studying profiles of rhythmic and non-rhythmic expression in the pineal gland.

## Results

### *In vivo* night/day differential expression of pineal transcripts, with and without neurotransmission

**Fig 1:**
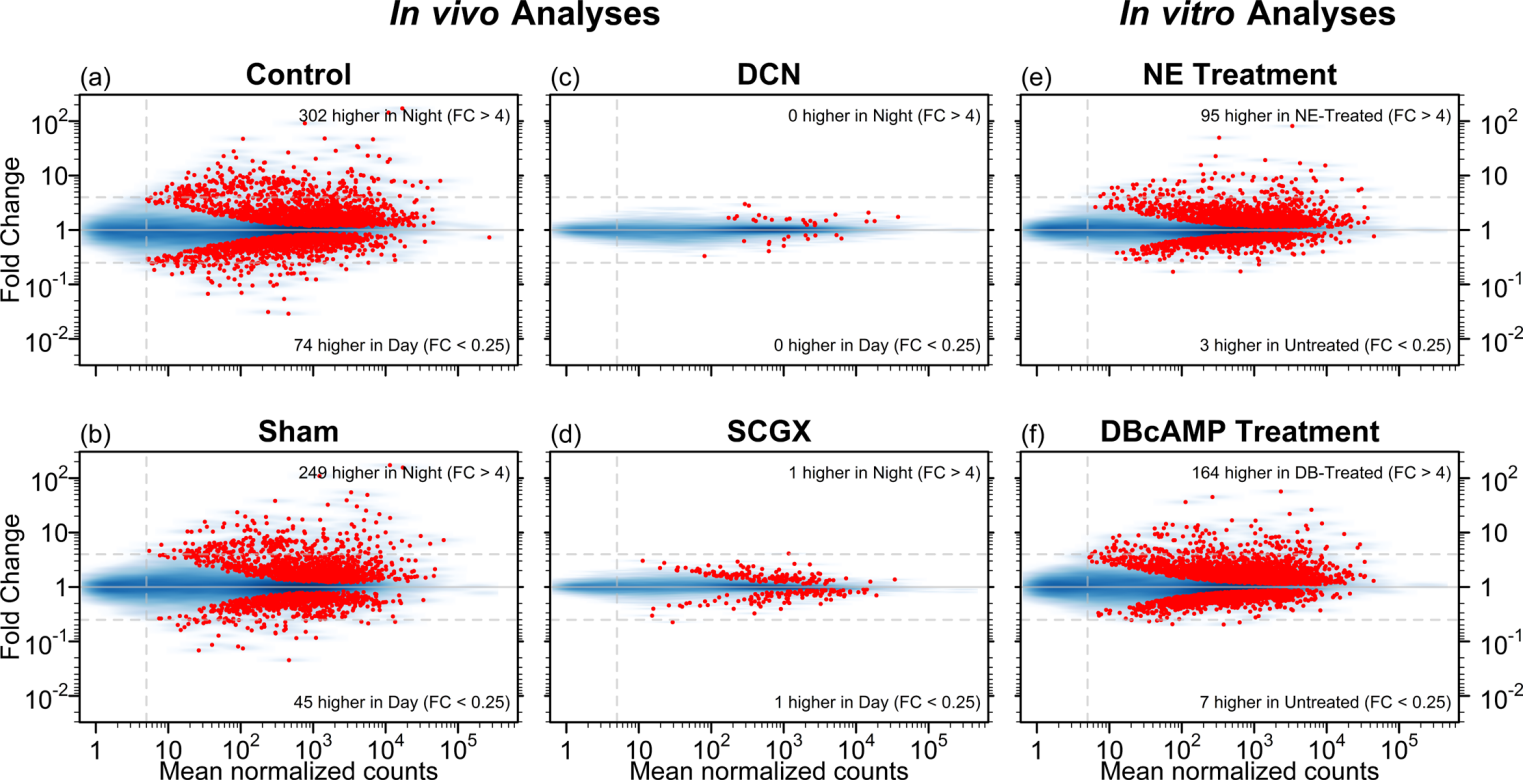
MA plots for six differential expression analyses. These plots display the mean normalized read-pair counts (x-axis) versus the estimated fold change (y-axis), on a log-log scale for four *in vivo* analyses and two *in vitro* analyses. The blue shading indicates the density of genes, and each red point represents a gene with statistically significant differential expression (adjusted-p < 0.001). Dashed horizontal lines mark 4-fold changes in both directions, dashed vertical line indicates minimum abundance threshold for the statistical tests. The four *in vivo* analyses compared night and day time points in adult rats for the following groups: (a) no surgery (Control); (b) neonatal sham surgery (Sham); (c) neonatal superior cervical ganglia decentralization (DCN); (d) neonatal superior cervical ganglionectomy (SCGX). The two *in vitro* analyses compared treated/untreated pineal glands: (e) norepinephrine-treated (NE) vs untreated and (f) dibutyryl-cyclic-AMP-treated (DBcAMP) vs untreated.

**Fig 2:**
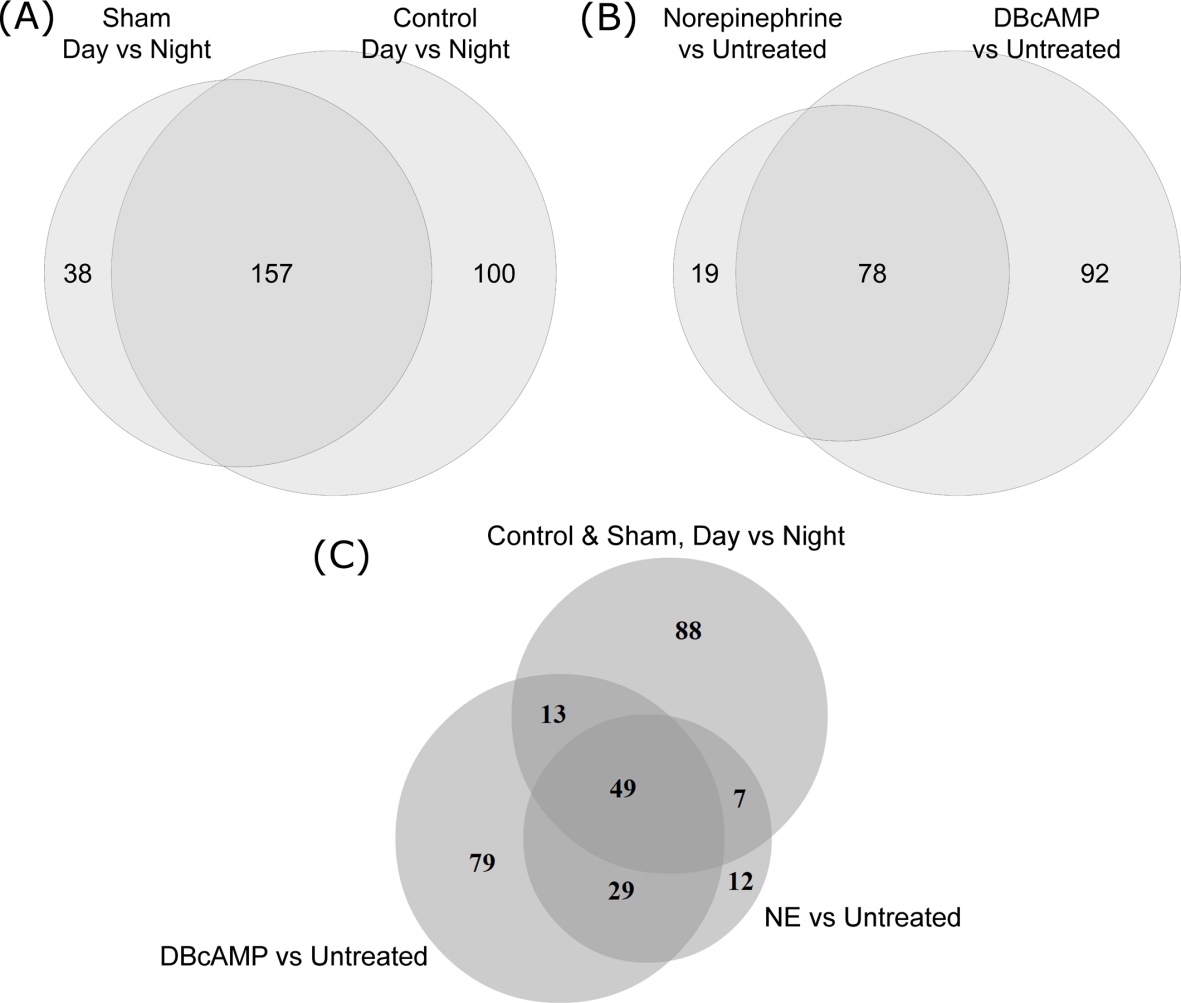
Venn diagrams displaying the overlap between the different differential expression analyses. Diagrams indicate the concordance in the genes found to display strong (FC > 4 in either direction) and statistically significant (adjusted-p < 0.001) differential expression in multiple separate analyses. (a) Comparison of the (*in vivo*) Control and Sham night/day differential expression analyses. (b) Comparison of the (*in vitro*) NE vs untreated and DBcAMP vs untreated differential expression analyses. (c) Comparison of the overlapping region from (a) with the results of the two *in vitro* analyses from (b).

More than 3000 genes showed statistically significant night/day differential expression in the Control and Sham animals at an adjusted-p-value cutoff of 0.001, with more than 1400 genes exhibiting greater than 2-fold changes in either direction (Fig 1a and 1b, Table 1). Of these, 257 genes exhibited strong differential expression in the Control group with fold change (FC) greater than 4 in either direction, 195 genes met these criteria in the Sham-operated group, and 157 genes exhibited met these criteria in both groups (Fig 2a). The RNA-Seq results replicated 255 out of the 343 genes found in previous microarray-based studies to exhibit greater than 2-fold night/day differential expression (in either direction) at the adjusted-p < 0.05 level (S1 Fig, S1 Dataset) [9]. In addition, more than a thousand genes were identified that had not been previously found to exhibit differential expression between night and day at this level.

**Table 1:** Genes significantly differentially expressed in the pineal gland on a night/day basis in Control and/or Sham groups, *in vivo* (adjusted-p < 0.001). Genes that exhibit strong differential expression in both are listed in bold (genes are classified by the least-extreme fold change across the two analyses). Note that some genes are listed twice if the fold changes of the two analyses fall in different intervals. In the SCGX and DCN groups, none of the genes displayed in this table exhibited night/day differential expression at this level (adjusted p < 0.001, FC > 4 in either direction). A complete list of all genes with fold changes, p-values, and normalized expression estimates is available in the SI (S1 Dataset).

Fold Change	Gene Symbol
**>32**	**Aanat** * ^†^, **Atp7b** * ^†^, Dclk3* ^†^, Dusp1^†^, Fcer1a* ^†^, Hcrtr1^†^, Hhip^†^, Irs2* ^†^, Nap1l5^†^, Ribc2* ^†^, **Sik1** * ^†^, **Slc15a1** ^†^
**16–32**	Cd24^†^, **CREM** * ^†^, **Dclk3** * ^†^, Dos^†^, **Drd4** * ^†^, **Dusp1** ^†^, **Fcer1a** * ^†^, **Hcrtr1** ^†^, **Irs2** * ^†^, **LOC100362205**, MGC94891*, **Nap1l5** ^†^, **nod3l** ^†^, **Pde10a** * ^†^, PORF1*, **Ptch1** * ^†^, Spata2L, St8sia2*, **Wnt10a** ^†^
**8–16**	**Accn2**, **B3gnt8** *, Bhlha15, **Brunol6**, Camk1g* ^†^, Ccrl2^†^, **Cd24** ^†^, **Chd5**, **Coq10b** * ^†^, **Cyp2s1**, D3ZJR7^†^, **Dclk1** ^†^, **Dio2** * ^†^, **Dos** ^†^, Elfn1, Errfi1^†^, Etnk1* ^†^, **F1LN23**, Fam160b1* ^†^, Gadd45b, Galntl1* ^†^, **Gem** * ^†^, **Gls2** ^†^, **Grm2**, Il20ra, Ipcef1, **Irak2** * ^†^, **Kcnq4** *, Kctd3* ^†^, Lmo1, LOC691317*, **Nr4a1** ^†^, **Nr4a3** ^†^, Osbpl6, Padi4* ^†^, Pde4b* ^†^, **Pde8b** ^†^, **Per2** ^†^, Pnma3, RGD1309903, RGD1310852*, RGD1560523* ^†^, RGD1561499, RGD1565158, Rgs2^†^, **Rxfp2** *, Slc17a6^†^, **Spata2L**, **St8sia2** *, **St8sia5** ^†^, **Syt4** ^†^, **Tinagl1** ^†^, **Zrsr1** * ^†^
**4–8**	7SK*, Actn2, Acy3, Ankrd52^†^, **ARLY** ^†^, **Arpp21** ^†^, Asgr1, **B3gnt2** ^†^, **Bex1** ^†^, **Bhlha15**, **Bsx** *, Cacna1h* ^†^, **Calhm1** *, Calhm2* ^†^, **Camk1g** * ^†^, **Ccrl2** ^†^, Cd8a^†^, Ceacam1, Ceacam10, Clstn3, Csrnp1, D3ZAQ3, **D3ZDX5** ^†^, **D3ZJR7** ^†^, D3ZRS0, D3ZW88* ^†^, D4A582, D4A5F4, **D4A8J6** *, D4ACZ5^†^, D4AE14^†^, **Dnm2** ^†^, **Drd1a**, **Egr1** ^†^, **Elfn1**, **Elfn2** ^†^, Ell2^†^, **EMAL5** *, **Emd** ^†^, **Ephx4**, **Errfi1** ^†^, **Etnk1** * ^†^, **F1LNL3** * ^†^, F1LNM2^†^, **F1LU85** ^†^, **F1LWE5**, F1LXI5, F1LY53, **F1M6I6**, F1M848*, **F1M8Z8**, **Fam160b1** * ^†^, Fam161a*, Farp2* ^†^, **Fdx1** ^†^, Fgd2, Fkbp5* ^†^, **Fry** ^†^, **Gadd45b**, Gal, **Galntl1** * ^†^, Gdf15* ^†^, **Glt8d3** * ^†^, **Gnaz** ^†^, **Gpr158**, **Greb1l** *, **Grm1** ^†^, Hspa1a^†^, Hspa5^†^, **Igf1r** ^†^, **Il20ra**, **Ipcef1**, Irf8^†^, **Itih1**, **Kcnv2**, **Kctd3** * ^†^, Krt19^†^, **Lamb3** ^†^, Lhx2, **Lmo1**, LOC100361460*, LOC100364604, LOC689856, **LOC691141**, **LOC691317** *, Lrp8, Lrrc43, **Lypd6b**, Mal2, Man2a1^†^, Mapk10^†^, Mapk4, **Mbnl2** * ^†^, **Mcam** ^†^, **Mettl8** *, Mfsd2, **MGC94891** *, Mmd^†^, Mmd2^†^, Mt1a^†^, Myh13*, **Ncald** ^†^, Neurog1*, Nos1, **Nptx1** * ^†^, **Osbpl6**, Padi1, **Padi4** * ^†^, **Parvb** ^†^, Pcdh1^†^, **Pde4b** * ^†^, **Pde7b**, Pdzrn3^†^, **Per1**, **Plagl1**, Plcxd1, Plk5* ^†^, **Pmepa1**, **Pnma3**, **PORF1** *, Ptp4a1^†^, **PVR** ^†^, **Q7TP44** ^†^, Rap1gap, **Rcan1** * ^†^, **Rem2**, RGD1309108* ^†^, RGD1309489, RGD1309586*, **RGD1309903**, **RGD1310852** *, **RGD1560523** * ^†^, **RGD1566021** *, **Rgs2** ^†^, **Rlim**, Rnase1*, Rorb*, Scnn1b, Shc3, Sla2, **Slc17a6** ^†^, Slc30a2^†^, **Smc1b**, **Spag4**, **Tbc1d1** ^†^, **Tbc1d30** ^†^, Tbx3, Thap4^†^, Thbs4, Thrb, Tmem108^†^, Tmem225, **Tpcn1** ^†^, **Trank1**, Trnp1, **Tyro3** ^†^, Wfikkn2*, **Xpot** ^†^
**1/4–1/8**	Adra1a, Akr1b8, Angptl4^†^, Atp10a, B4F7B7, Barhl2, Bpil3, Cacna1e, Car8, **Ccdc85a**, **Ccl9** ^†^, Chrna5, Cpt1a^†^, Crp, Crybg3, Cstl1*, **D3ZV53** *, **D3ZW53** *, **D3ZX30**, **D3ZXI1**, D3ZYL1, **D4A0N6**, D4A1L0, Dhrs9, Dscaml1, F1LUT6, **F1LVH8**, F1LW34, F1M0S2, **F1M1I4** *, **F1M7W8**, Frmd4b, **Fscn2**, **Galnt12**, Galnt14, Gata1*, Gng11, Gramd2, Grip2^†^, Gucy1a3^†^, Hook1, **Hs3st2** * ^†^, **Htra4**, IGG2B, Il17re, **Lad1-ps1**, **LOC56764**, **LOC690918** *, Ms4a2, Ms4a3, Ndrg1^†^, **Npy1r** ^†^, Opn1sw* ^†^, Opn3, **Prc1** ^†^, Pter, RGD1308116^†^, RGD1308133, RGD1310773, RGD1559884*, RGD1560925, **RGD1563092**, RGD1564942, **Rgs20**, Rnase1l2*, Slc37a2, Slc39a12, Slc6a13, Slco4c1, Sstr3^†^, **St8sia3** ^†^, Sult1c3, **Tfpi2** ^†^, Ucp3, **Wdr89** ^†^, Zfp385d
**1/8–1/16**	Ccl9^†^, D3ZW53*, F1M7W8, Fbp2, Fscn2, Hs3st2* ^†^, Htra4, HYES, LOC100362965, LOC100365668, LOC690918*, Npy1r^†^, **PTN21** ^†^, **RGD1304931**, **Rptn** *
**1/16–1/32**	Fam111a, **RGD1304963** ^†^
**<1/32**	RGD1304963^†^

*Gene exhibits high relative expression in the pineal gland (see S2 Table).

^†^Gene has previously been found to be differentially expressed between day and night conditions in microarray experiments.

Stimulus deprivation resulting from neonatal SCGX or DCN eliminated nearly all night/day differential changes observed in the Control- and Sham-operated groups (see Fig 1c and 1d). Principal component analysis reveals that the SCGX and DCN samples for both day and night cluster tightly with the Sham-day and Control-day samples (S2e Fig). Furthermore, when the SCGX or DCN groups were directly compared with the Sham group, the vast majority of the differences were found at night in rhythmic genes (S2a–S2d Fig). All of these observations are consistent with the hypothesis that the changes resulting from SCGX and DCN are primarily due to the loss of SCG-mediated central neural stimulation.

A small set of genes were identified in which the expression differed significantly (adjusted-p < 0.001), substantially (FC > 2 in either direction), and consistently (FC in the same direction at both day and night) between the DCN and Sham groups (Table 2, S2 Dataset). Of particular note: at both day and night the gene Serpina1 exhibited a >6-fold reduction in expression in the DCN group compared with the Sham group. Weaker but still significant effects were observed in Serpina1 when comparing the SCGX group to the Sham group.

**Table 2:** Genes with consistent differential expression in the DCN group relative to the Sham group. These genes displayed significant (adjusted-p < 0.001) differentials across 2 analyses: DCN-day vs Sham-day and DCN-night vs Sham-night. Genes are classified by the least-extreme fold change across both analyses. The complete results for these analyses are available in the SI (S2 Dataset).

Fold Change	Gene Symbol
**>8**	-
**4–8**	Rcvrn, LOC681316, RGD1304731
**2–4**	Gabra1, F1LZ56, RGD1306717, Kcnab1^†^, E9PSL1, March4, Cldn7, Rnf182, Ppp1r3c, Tuft1, Kcnj11, ABCC8, Rnf222*, D4A4G4, RGD1563692, RGD1563070, Gata5, LOC688006
**1/2–1/4**	Fry^†^ ^‡^, Lnx1^†^, Parm1^§^, Wfikkn2* ^‡^, F1LVN4, Pde4b* ^†^ ^‡^ ^§^, RGS6, Gngt1, F1LWK7, Scg2^†^, D3ZEA8, Ptprn*, Prr16, F1MAG9*, Sult2b1^†^, LOC691300^§^, Rdh2, RGD1563903*, Dclk3* ^†^ ^‡^ ^§^, CAD23, Gpr123, Glod5, Cpe
**1/4–1/8**	Sftpa1, Rln3* ^§^, Pde10a* ^†^ ^‡^ ^§^, Il20ra^‡^, Gda, Tyrp1, Serpina1, Ribc2* ^†^ ^‡^ ^§^
**<1/8**	-

*Gene exhibits high relative expression in the pineal gland (see S2 Table).

^†^Gene has previously been found to be differentially expressed between day and night conditions in microarray experiments (adjusted-p < 0.05).

^‡^Gene has strong (FC > 4 or FC < 0.25) and statistically significant (adjusted-p < 0.001) differential expression in the Control and/or Sham night/day *in vivo* analyses (Table 1).

^§^Gene has strong and statistically significant differential expression in either or both of the treated vs untreated *in vitro* analyses (Table 3).

Three genes dedicated to melatonin synthesis: Asmt, Tph1, and Gch1, were not found to exhibit strong changes in expression as a result of SCGX or DCN. In comparisons between the SCGX/DCN groups with the Sham group at day and night, these three appeared to consistently differ by less than 35%. While some of these differentials were moderately statistically significant (adjusted-p < 0.05), the very small effect size implies that neural stimulation is not required to maintain relatively-high levels of expression.

### *In vitro* differential expression of pineal transcripts resulting from treatment with norepinephrine or dibutyryl cyclic AMP

As indicated in the introduction, neural control of pineal transcription involves release of norepinephrine and elevation of the second messenger cyclic AMP. This was extended using pineal organ culture. Glands were incubated for 48 hours under control conditions and then treated with norepinephrine or dibutyryl cyclic AMP. Subsequent RNA-Seq analysis found that 97 genes displayed strong and statistically significant differential expression in the norepinephrine-treated vs untreated analysis, and 170 in the dibutyryl-cyclic-AMP-treated vs untreated analysis (adjusted-p < 0.001, FC > 4 in either direction, see Fig 1e and 1f). A set of 78 genes met these criteria in both analyses (see Table 3, Fig 2b, S3 Dataset).

**Table 3:** Genes significantly differentially expressed in pineal gland tissues in response to *in vitro* treatment with NE or DBcAMP (adjusted-p < 0.001). Genes that exhibit strong differential expression in both the NE and DBcAMP analyses are listed in bold (genes are classified by the least-extreme fold change across the two analyses). A complete list of all genes with fold changes, p-values, and normalized expression estimates is available in the SI (S3 Dataset).

Fold Change	Gene Symbol
**>32**	**Aanat** * ^†^, RGD1305627, **Slc15a1** ^†^
**16–32**	Dclk3* ^†^, **Dusp1** ^†^, Fcer1a* ^†^, Il11, Nr4a1^†^, Rgs2^†^
**8–16**	**Actn2**, Atp7b* ^†^, Bhlha15, Calca, Ccrn4l, **Cd24** ^†^, **Chd5**, Chst2^†^, CREM* ^†^, D4A599, **Dclk3** * ^†^, Dct, **F1LWE5**, Fam148b, **Fcer1a** * ^†^, Giot1, Gpd1, Grm2, Has1, Hcrtr1^†^, Il6, **Ipcef1**, **Irs2** * ^†^, Kcnk10, Kcnq4*, LOC100360880, LOC100361460*, LOC691300, Mstn, **Mustn1**, Nap1l5^†^, Nefh, Nlrp10, Nmu, **Nptx1** * ^†^, **Nr4a1** ^†^, Nr4a3^†^, **Pde10a** * ^†^, **Pdp2** ^†^, Plagl1, **Ptch1** * ^†^, **Rgs2** ^†^, **Ribc2** * ^†^, Rln3*, Rrad, **Sik1** * ^†^, Slc17a6^†^, Sstr2, Tinagl1^†^, Vgf
**4–8**	Adamts15, **Ankrd13b**, **Atp7b** * ^†^, B3gnt2^†^, **B3gnt8** *, **Bhlha15**, Bsx*, **Camk1g** * ^†^, Cbln1, **Ccrn4l**, **Chst2** ^†^, Cited1*, Clic6, Clstn3, **Coq10b** * ^†^, Cpd, **CREM** * ^†^, Csf3, Cxcl3, Cxcl6, D3ZD52, D3ZHM0, D3ZVN6, **D3ZW88** * ^†^, D4AA43, **Dos** ^†^, Drd4* ^†^, Errfi1^†^, **Etnk1** * ^†^, F1LU85^†^, F1LXI5, F1LZR9, F1M1S6, F1M578, F1M8Z8, Fam107b, Fam160b1* ^†^, Fam46b, Fam71e2, **Fdx1** ^†^, **Fgd2**, Fos, Gadd45b, Gchfr, **Gem** * ^†^, **Ggcx** *, **Giot1**, Gjc2, Glt8d3* ^†^, **Gpd1**, Gpr83, Gprc5a, Greb1l*, **Grm2**, **Hcrtr1** ^†^, Herc4, Hhip^†^, Id1^†^, Il1b, Il1rn, Il22, Inhba, **Irak2** * ^†^, **Kcnq4** *, Klf4, **Lamb3** ^†^, LOC100360507, **LOC100360880**, **LOC100361460** *, LOC681749, **LOC691300**, Lrrn3^†^, Lxn^†^, Mal2, Map3k8, Masp1, Mfsd2, **MGC94891** *, Mmd2^†^, **Mstn**, Musk*, Myo16, **Nap1l5** ^†^, **Nmu**, Nod2, **nod3l** ^†^, Nos1, Nos2, Npas4, Nppc, Nr4a2, **Nt5dc3**, Nt5e, **Osbpl6**, **Padi4** * ^†^, **Parm1**, **Pde4b** * ^†^, Pde8b^†^, Pdxk, Plk3, Prickle1, **Prokr2**, Prrg4, Ptp4a1^†^, Q6MG91, Q7TP44^†^, Rasd1, Rassf8, Rcan1* ^†^, **Rem2**, **RGD1560523** * ^†^, RGD1564664, RGD1566021*, RGD1566035, Rhobtb3, Rlim, **Rln3** *, **Rrad**, Rxfp1, Rxfp2*, Scnn1b, **Scnn1g**, Sgk493, **Slc16a6**, **Slc17a6** ^†^, Slc23a2^†^, **Slc25a25**, **Slc30a2** ^†^, Slc30a3, Sostdc1, Spt1, **Sstr2**, **St8sia2** *, Syt13, **Syt4** ^†^, **Tbc1d1** ^†^, Thbd, **Tinagl1** ^†^, **Tkt**, Top1, **Wnt10a** ^†^, **Xpot** ^†^, Yod1, **Zrsr1** * ^†^
**1/4–1/8**	F1M1I4*, Klkb1, Lgi2, **LOC690918** *, PTN21^†^, **RGD1308133**, Slitrk6, Wnt2b*
**<1/8**	-

*Gene exhibits high relative expression in the pineal gland (see S2 Table).

^†^Gene has previously been found to be differentially expressed between day and night conditions in microarray experiments.

Interestingly, even though the Serpina1 gene displays no apparent night/day differential expression *in vivo*, it does display a moderate increase in expression *in vitro* when treated with NE (adjusted-p = 0.00001, FC = 2.61) or dibutyryl cyclic AMP (adjusted-p = 0.030, FC = 1.73) (S3 Dataset).

Comparison of the *in vivo* and *in vitro* experiments revealed strong concordance between the *in vivo* Control and Sham-operated day/night experiments and both *in vitro* treated/untreated experiments, with 49 genes exhibiting strong (FC > 4 in either direction) and highly-significant (adjusted-p < 0.001) differential expression in all four analyses (see Fig 2c; S1 Table).

### Identification of pineal marker genes

Comparison of the abundance of transcripts in the pineal gland (at both day and night) with other tissues identified a set of 349 strongly-expressed genes (normalized read-pair count > 30) with a maximum-likelihood fold change greater than 8 in the pineal gland at either day or night (S3 Fig, S2 Table, S4 Dataset). Due to the unavailability of biological replicates in this comparison, hypothesis tests could not be performed on these comparisons. Thus, in the absence of other corroborative evidence (such as data from other studies [9]), genes identified as being highly expressed in the pineal gland by this analysis alone are best viewed as potential candidates for further investigation using other experimental approaches.

## Discussion

This effort has expanded our knowledge of the impact that neural stimulation has on the pineal transcriptome by identifying regulated genes that are expressed differentially on a 24-hour schedule. Perhaps the most remarkable element is the requirement of sympathetic neural stimulation for essentially all of the observed 24-hour changes in thousands of pineal transcripts. This adds to evidence that the transcriptome profile can be dynamically regulated by neurotransmission, while providing little-to-no support for the view that peripheral mechanisms operating within the rat pineal gland play an important role in the night/day changes in gene expression in this tissue, a topic that deserves further study.

In the case of the pineal gland, it is likely that many of the observed changes occur in pinealocytes, which constitute more than 95% of the cells in the rodent pineal gland [39]. However, it is also possible that some of the observed changes occur in other resident cell types including interstitial cells, endothelial cells, glia cells, or in transient cells. Establishing the precise localization of the observed changes in transcript abundance is important in understanding the functional relevance of the observed changes.

Previous analyses of the daily changes in the pineal transcriptome have made it clear that essentially all aspects of cell physiology are subject to neural control [9]. It would appear that this reflects the dedication of the pineal gland to the daily production of melatonin. Although one of the enzymes in this pathway is under neural control, such control appears to involve processes with no obvious link to melatonin production, including many unrelated functions linked to lipid biology, small molecule transport, extracellular matrix processing, cell-cell contact and other processes.

### Transsynaptic control of the pineal transcriptome

It is well-established from previous studies that production of melatonin is neurally controlled via the norepinephrine/cyclic AMP mechanism [9]. In this study we extended the observation that many of the changes in transcript abundance that occur at night are reproduced *in vitro* by treatment with either norepinephrine or dibutyryl cyclic AMP. This included the transcription factor encoding genes Nr4a1 and Nr4a3 which were found in previous studies to increase at night and in response to norepinephrine [9, 40]. Thus, this adds to evidence that these transcription factors are likely to mediate cyclic AMP control of the pineal transcriptome. All of these observations provide strong support for the hypothesis that neural stimulation via norepinephrine drives the bulk of the observed changes in the pineal transcriptome. However, the results also provide reason to consider that some neurally induced changes seen *in vivo* on a night/day basis are not simply a reflection of the presence of norepinephrine acting through cyclic AMP. Many genes with observed *in vivo* night/day differentials did not exhibit strong and statistically significant differential between untreated and treated samples, *in vitro*. There are several possible hypothetical explanations for this. First of all, the statistical methods used here do not guarantee uniform power between experiments and such apparent discrepancies can occur by chance. Secondly, it is possible that the pineal gland is less responsive in organ culture because *in vitro* conditions do not optimally reproduce *in vivo* conditions, and (undetermined) essential factors may be absent from the defined culture medium. This phenomenon has been observed in the pineal gland with the adrenergic stimulation of Drd4 expression, which is known from previous studies to require the addition of thyroid hormone [41]. Similar dependencies may exist for other genes. Thirdly, it is possible that these apparent inconsistencies between night/day and treated/untreated differentials may be due to the involvement of other transmitters. One candidate for this role is acetylcholine, which might be released via either central or peripheral cholinergic innervation of the pineal gland and act through highly-expressed nicotinic acetylcholine receptors [42]. Similarly, neuropeptide Y might play a role; it is present in sympathetic nerves innervating the pineal gland and could act through the neuropeptide Y receptor 1 [43, 44].

Furthermore, the observation that many of the genes induced by norepinephrine are also induced by dibutyryl cyclic AMP treatment supports the view that the effects of norepinephrine are mediated by cyclic AMP. This observation does not, however, preclude the possibility that second messengers other than cyclic AMP might be required for norepinephrine to control the abundance of some genes. Candidates for this role include Ca^++^, cyclic GMP and phospholipids, all of which are controlled by norepinephrine and could modulate cyclic AMP induction of genes [25, 45–47].

### The effects of SCGX and DCN: Similarities and Differences

The ganglionectomy and decentralization surgical procedures were expected to produce somewhat similar phenotypes, as both eliminate or at least greatly reduce the rhythmic stimulation of the pineal gland. By comparing and contrasting these groups with one another and with the sham-surgery group we were able to identify previously unidentified subtleties in the regulation of the pineal transcriptome.

Our analyses detected more statistically significant night/day differentially expressed genes in the SCGX animals than in the DCN animals. This apparent difference could be a coincidence, or could be due to the effects of circulating catecholamines, which gain access to pinealocytes in the absence of sympathetic nerves in the pineal gland of the SCGX animals. The sympathetic innervation of the pineal gland, responsible for uptake of extracellular catecholamines, is eliminated by SCGX, but remains intact in DCN animals, thereby preventing stimulation by these circulating catecholamines [32].

Our comparisons of the DCN and SCGX animals to the sham-surgery animals suggest that the neonatal interruption of neural stimulation does not broadly alter the pineal transcriptome other than preventing rhythmic changes in gene expression. The principal component analysis and the cross-group analyses demonstrated that any differences in the pineal transcriptome resulting from developmental changes in the SCGX or DCN animals are (at worst) minor in comparison to the profound changes that occur between night and day conditions in intact rats (S2 Fig, S2 Dataset). In addition, we found only small changes of three pineal marker genes, Tph1, Asmt, and Gch1, indicating that neural stimulation is not required to maintain these outstanding features of the pineal transcriptome during post-neonatal development.

An interesting observation was made in this study regarding Serpina1, a member of the Serpin family of peptidase inhibitors, which has been well studied biochemically and in human disease [48, 49]. This gene was downregulated following SCGX and DCN, and was upregulated following treatment with norepinephrine or dibutyryl cyclic AMP. This, together with the knowledge that it is a secreted protease inhibitor, provides reason to speculate that the encoded protein mediates neurotransmission-dependent control of the extracellular matrix by inhibiting extracellular proteases, as is the case for another serpin family member, neuroserpin [49, 50]. Serpina1 might mediate neural regulation of cell-cell attachments, sympathetic synaptic architecture, features of vascularization, or some combination thereof.

### Impact on future investigations

The results presented here provide a number of valuable resources for future investigators. Tables in the SI provide the abundances and fold changes for all annotated genes and for all analyses. This includes genes exhibiting strong and statistically significant *in vivo* night/day differential expression and those exhibiting strong and statistically significant *in vitro* treated/untreated differential expression. Additionally, the SI provides data comparing the abundance of each transcript in the pineal gland to average expression in other tissues. These resources could be useful in identifying potentially productive targets for further study.

## Methods

### *In vivo* night/day differential expression of pineal transcripts

To identify and characterize rhythmic transcripts and to study the effects of surgical stimulus deprivation *in vivo*, pineal gland samples were taken from adult rats in each of four groups and each of two time points. The four surgical groups were: no surgical intervention (Control); neonatal sham surgery (Sham); neonatal bilateral SCG decentralization (DCN); neonatal bilateral superior cervical ganglionectomy (SCGX) [21]. Each surgical group had 18 to 20 animals, which were then divided into two sub groups which were euthanized at mid-day (ZT7) or midnight (ZT19). Samples were pooled into three biological replicates for each of the eight biological conditions (24 pooled samples, total).

### *In vitro* differential expression of pineal transcripts resulting from treatment with norepinephrine or cyclic AMP

To further elucidate the relationship between innervation and transcript regulation, a second experiment was run using pineal gland organ culture[51]. Three treatment conditions were compared: untreated control (Untreated), norepinephrine-treated (NE), and dibutyryl-cyclic-AMP-treated (DBcAMP). For each of the three treatment conditions, samples were pooled into three biological replicates (9 pooled samples, total).

### Identification of pineal marker genes

To identify transcripts that are highly expressed in the pineal gland relative to other tissues, pineal gland samples were taken at mid-day (ZT7) or midnight (ZT19). Additional samples were taken from the following tissues at ZT7: cortex, cerebellum, midbrain, hypothalamus, hindbrain, spinal cord, retina, pituitary, heart, liver, lung, kidney, skeletal muscle, small intestine, and adrenal gland. Total RNA was extracted from each tissue, and then equal amounts of each of the 15 tissues were combined for the final pooled “mixed-tissue” sample. Three pooled samples were created: one each for pineal-day, pineal-night, and mixed-tissue (3 pooled samples, total).

### Animals

Sprague Dawley rats (Taconic Farms, Germantown, NY) were used for these studies and were maintained on a 14:10 light:dark (L:D) cycle.

For the *in vivo* differential expression experiments, timed pregnant animals were used; on postnatal day 5 male and female pups were divided into the four surgical groups and subjected to the respective surgical interventions. Animals were then maintained in a 14:10 L:D cycle until day 40. For the *in vitro* experiments, female rats were used that had been maintained for at least two weeks in a 14:10 L:D cycle before being euthanized (2 to 3 months old).

Animals were euthanized by CO_2_ asphyxiation and decapitated. Animals sacrificed at ZT19 were decapitated in dim red light. Pineal glands were immediately placed in microtubes on solid CO_2_ (three glands per tube); samples of other tissues (approximately 10 mg) were rapidly prepared and placed in individual tubes. Tissues were stored at -80°C until use.

Animal use and care protocols were approved by the NIH Institutional Animal Care and Use Committee and followed the guidelines of the National Research Council's Guide for Care and Use of Laboratory Animals (Vol. 8) and the Animal Research: Reporting *In vivo* Experiments (ARRIVE) guidelines.

### Surgical procedures

The neonatal DCN and SCGX were based on modifications to previously published procedures for adult rats (See SI) [21].

### Organ culture

For the *in vitro* experiments, pineal glands were cultured as described [51] for a period of 48 hours under control conditions, then treated with norepinephrine (NE; 1 μM) or dibutyryl cyclic AMP (DBcAMP; 500 μM), or left untreated (CN) for 6 hours before being harvested into microtubes placed on solid CO_2_ (three glands per tube).

### Sample preparation and sequencing

RNA was prepared from pools of pineal glands and from samples of “mixed-tissue” RNA as detailed in the SI.

To minimize sequencing batch effects all samples from each experiment were barcoded and pooled into combined libraries. The strand-specific pooled libraries were sequenced on a HiSeq2000 (Illumina, San Diego, CA) using version 3 chemistry. The resultant reads were aligned with the RNA-STAR aligner, using the rn4/RGSC3.4 genome assembly and Ensembl transcript annotation, release 69 [52, 53]. Quality control metrics were calculated and visualized using the QoRTs software package, and no major artifacts or abnormalities were found [54]. Additional details concerning the sequencing, library preparation, and alignment are described in the SI.

### Differential expression analysis

Gene read counts were provided by QoRTs, using the same algorithm defined by the HTSeq package [55]. Differential expression analysis was performed using DESeq2 [56]. In each analysis, genes with a mean normalized read-pair count of less than 5 were not tested.

The pineal marker gene analysis followed a slightly different methodology. Two separate comparisons were run: pineal-day vs mixed-tissue and pineal-night vs mixed-tissue. These comparisons were run without biological replicates, and thus the biological dispersion could not be rigorously estimated and no statistical hypothesis tests could be performed. Instead, the maximum likelihood fold changes were calculated between the pineal and mixed tissue samples using DESeq2. Gene lists were generated using simple thresholds on the mean read-pair count and the maximum likelihood fold changes between the pineal and mixed-tissue samples. We selected a normalized read-pair count threshold of 30 and a fold change threshold of 8.

## Supporting Information

S1 Appendix Supplementary methods. (DOCX)

S1 Dataset Complete results tables for genes differentially expressed in the pineal gland on a night/day basis, *in vivo*. (XLSX)

S2 Dataset Complete results tables for the SCGX/DCN vs Sham analyses. (XLSX)

S3 Dataset Complete results tables for genes differentially expressed in pineal gland in response to *in vitro* treatment with norepinephrine or dibutyryl cyclic AMP. (XLSX)

S4 Dataset Complete results tables for mixed-tissue vs pineal-gland comparisons. (XLSX)

S1 Fig Comparison of gene lists detected by the Control day-vs-night RNA-Seq analysis and a previous microarray-based analysis. Gene lists were compiled using an adjusted-p-value threshold of 0.05 and a fold change threshold of 2 (in either direction). *Note: 22 gene names that were found in the microarray results could not be matched with any current or former gene name of any known gene name in the Ensembl (release 69) or RGD (version 6) databases. Additionally: 155 microarray probe loci met the significance/fold-change criteria, but were not annotated with any known gene. These genes/probes were not counted in the figure above.
(TIF)

S2 Fig Summary plots for the cross-group analyses, in which SCGX and DCN groups were compared with the Sham group at both day and night. (a-d) MA plots for all four comparisons. Statistically significant genes (adjusted-p < 0.001) are marked with dots, colored based on their night/day differential status from the Control night/day analysis: blue indicates that the gene is upregulated at night, red indicates that it is downregulated at night, black indicates that the gene is not significantly differentially expressed between night and day (adjusted-p > 0.05). The displayed counts indicate the number of dots of each color above or below the FC > 2 or FC < 0.5 thresholds. A complete list of all genes with fold changes, p-values, and normalized expression estimates is available in the SI (S3 Dataset). (e) Plot of the first two principal components from a principal component analysis across all 24 *in vivo* samples. Note that based on the first principal component, the Control-Night and Sham-Night samples cluster strongly to the right, while all other samples (including the SCGX and DCN samples for both day and night) cluster strongly to the left.
(TIF)

S3 Fig MA plot of the comparisons between mixed tissue with pineal glands at both day and night. Genes were marked in red if the mean normalized read-pair count exceeded 30 and if the (maximum-likelihood) fold change was greater than 8. Note that due to the lack of replicates no significance p-values could be calculated.
(TIF)

S1 Table Genes exhibiting differential expression in both *in vivo* and *in vitro* experiments. The genes listed exhibit statistically significant (adjusted-p < 0.001) differentials in all of the following four comparisons: Control night vs day, Sham night vs day, untreated vs NE-treated, and untreated vs DBcAMP-treated.
(DOCX)

S2 Table Genes with relatively high expression in the pineal gland. Gene symbols (when available) of all genes with > 32-fold enrichment in the pineal gland (day and/or night) and mixed non-pineal tissues. Genes that exhibit high relative expression in both the day and night analyses are listed in bold (based on the minimum of the two fold changes). A more complete list is available in the SI (S4 Dataset).
(DOCX)

## References

[1] Bustos DM, Bailey MJ, Sugden D, Carter DA, Rath MF, Moller M, et al Global daily dynamics of the pineal transcriptome. Cell and tissue research. 2011;344(1):1–11. Epub 2011/02/09. 10.1007/s00441-010-1094-1 . 21302120

[2] Klein DC, Bailey MJ, Carter DA, Kim JS, Shi Q, Ho AK, et al Pineal function: impact of microarray analysis. Molecular and cellular endocrinology. 2010;314(2):170–83. Epub 2009/07/23. 10.1016/j.mce.2009.07.010 19622385 PMC3138125

[3] Karolczak M, Korf HW, Stehle JH. The rhythm and blues of gene expression in the rodent pineal gland. Endocrine. 2005;27(2):89–100. Epub 2005/10/12. 10.1385/ENDO:27:2:089 . 16217122

[4] Maronde E, Stehle JH. The mammalian pineal gland: known facts, unknown facets. Trends in endocrinology and metabolism: TEM. 2007;18(4):142–9. Epub 2007/03/22. 10.1016/j.tem.2007.03.001 . 17374488

[5] Humphries A, Klein D, Baler R, Carter DA. cDNA array analysis of pineal gene expression reveals circadian rhythmicity of the dominant negative helix-loop-helix protein-encoding gene, Id-1. Journal of neuroendocrinology. 2002;14(2):101–8. Epub 2002/02/19. . 11849369 10.1046/j.0007-1331.2001.00738.x

[6] Roseboom PH, Coon SL, Baler R, McCune SK, Weller JL, Klein DC. Melatonin synthesis: analysis of the more than 150-fold nocturnal increase in serotonin N-acetyltransferase messenger ribonucleic acid in the rat pineal gland. Endocrinology. 1996;137(7):3033–45. Epub 1996/07/01. 10.1210/endo.137.7.8770929 . 8770929

[7] Baler R, Klein DC. Circadian expression of transcription factor Fra-2 in the rat pineal gland. J Biol Chem. 1995;270(45):27319–25. Epub 1995/11/10. . 7592994 10.1074/jbc.270.45.27319

[8] Klein DC, Coon SL, Roseboom PH, Weller JL, Bernard M, Gastel JA, et al The melatonin rhythm-generating enzyme: molecular regulation of serotonin N-acetyltransferase in the pineal gland. Recent progress in hormone research. 1997;52:307–57; discussion 57–8. Epub 1997/01/01. . 9238858

[9] Bailey MJ, Coon SL, Carter DA, Humphries A, Kim JS, Shi Q, et al Night/day changes in pineal expression of >600 genes: central role of adrenergic/cAMP signaling. J Biol Chem. 2009;284(12):7606–22. Epub 2008/12/24. 10.1074/jbc.M808394200 19103603 PMC2658055

[10] Klein DC, Weller JL, Moore RY. Melatonin metabolism: neural regulation of pineal serotonin: acetyl coenzyme A N-acetyltransferase activity. Proceedings of the National Academy of Sciences of the United States of America. 1971;68(12):3107–10. Epub 1971/12/01. 4332009 10.1073/pnas.68.12.3107 PMC389600

[11] Bowers CW, Dahm LM, Zigmond RE. The number and distribution of sympathetic neurons that innervate the rat pineal gland. Neuroscience. 1984;13(1):87–96. Epub 1984/09/01. . 6493487 10.1016/0306-4522(84)90261-6

[12] Bowers CW, Zigmond RE. The influence of the frequency and pattern of sympathetic nerve activity on serotonin N-acetyltransferase in the rat pineal gland. The Journal of physiology. 1982;330:279–96. Epub 1982/09/01. 7175744 10.1113/jphysiol.1982.sp014341 PMC1225298

[13] Lingappa JR, Zigmond RE. A histochemical study of the adrenergic innervation of the rat pineal gland: evidence for overlap of the innervation from the two superior cervical ganglia and for sprouting following unilateral denervation. Neuroscience. 1987;21(3):893–902. Epub 1987/06/01. . 3627441 10.1016/0306-4522(87)90045-5

[14] Klein DC. Photoneural regulation of the mammalian pineal gland. Ciba Foundation symposium. 1985;117:38–56. Epub 1985/01/01. 3015512 10.1002/9780470720981.ch4

[15] Yanovski J, Witcher J, Adler N, Markey SP, Klein DC. Stimulation of the paraventricular nucleus area of the hypothalamus elevates urinary 6-hydroxymelatonin during daytime. Brain research bulletin. 1987;19(1):129–33. Epub 1987/07/01. . 3651836 10.1016/0361-9230(87)90175-4

[16] Klein DC, Smoot R, Weller JL, Higa S, Markey SP, Creed GJ, et al Lesions of the paraventricular nucleus area of the hypothalamus disrupt the suprachiasmatic leads to spinal cord circuit in the melatonin rhythm generating system. Brain research bulletin. 1983;10(5):647–52. Epub 1983/05/01. . 6307491 10.1016/0361-9230(83)90033-3

[17] Klein DC, Moore RY. Pineal N-acetyltransferase and hydroxyindole-O-methyltransferase: control by the retinohypothalamic tract and the suprachiasmatic nucleus. Brain research. 1979;174(2):245–62. Epub 1979/10/05. . 487129 10.1016/0006-8993(79)90848-5

[18] Moore RY, Klein DC. Visual pathways and the central neural control of a circadian rhythm in pineal serotonin N-acetyltransferase activity. Brain research. 1974;71(1):17–33. Epub 1974/05/10. . 4595289 10.1016/0006-8993(74)90188-7

[19] Moga MM, Moore RY. Organization of neural inputs to the suprachiasmatic nucleus in the rat. The Journal of comparative neurology. 1997;389(3):508–34. Epub 1997/12/31. . 9414010 10.1002/(sici)1096-9861(19971222)389:3<508::aid-cne11>3.0.co;2-h

[20] Klein DC, Moore RY, Reppert SM. Suprachiasmatic nucleus: the mind's clock. New York: Oxford University Press; 1991 xvi, 467 p. p.

[21] Savastano LE, Castro AE, Fitt MR, Rath MF, Romeo HE, Munoz EM. A standardized surgical technique for rat superior cervical ganglionectomy. J Neurosci Methods. 2010;192(1):22–33. Epub 2010/07/20. 10.1016/j.jneumeth.2010.07.007 . 20637235

[22] Ho AK, Chik CL. Post-receptor mechanism in dual receptors regulation of second messengers in rat pineal gland. Progress in clinical and biological research. 1990;342:139–45. Epub 1990/01/01. . 2166288

[23] Ho AK, Chik CL. Modulation of Aanat gene transcription in the rat pineal gland. Journal of neurochemistry. 2010;112(2):321–31. Epub 2009/10/29. 10.1111/j.1471-4159.2009.06457.x . 19860854

[24] Klein DC, Sugden D, Weller JL. Postsynaptic alpha-adrenergic receptors potentiate the beta-adrenergic stimulation of pineal serotonin N-acetyltransferase. Proceedings of the National Academy of Sciences of the United States of America. 1983;80(2):599–603. Epub 1983/01/01. 6132380 10.1073/pnas.80.2.599 PMC393427

[25] Vanecek J, Sugden D, Weller J, Klein DC. Atypical synergistic alpha 1- and beta-adrenergic regulation of adenosine 3',5'-monophosphate and guanosine 3',5'-monophosphate in rat pinealocytes. Endocrinology. 1985;116(6):2167–73. Epub 1985/06/01. 10.1210/endo-116-6-2167 . 2986940

[26] Sugden D, Vanecek J, Klein DC, Thomas TP, Anderson WB. Activation of protein kinase C potentiates isoprenaline-induced cyclic AMP accumulation in rat pinealocytes. Nature. 1985;314(6009):359–61. Epub 1985/03/03. . 2984573 10.1038/314359a0

[27] Maronde E, Pfeffer M, von Gall C, Dehghani F, Schomerus C, Wicht H, et al Signal transduction in the rodent pineal organ. From the membrane to the nucleus. Advances in experimental medicine and biology. 1999;460:109–31. Epub 2000/05/16. . 10810507 10.1007/0-306-46814-x_14

[28] Simonneaux V, Ribelayga C. Generation of the melatonin endocrine message in mammals: a review of the complex regulation of melatonin synthesis by norepinephrine, peptides, and other pineal transmitters. Pharmacological reviews. 2003;55(2):325–95. Epub 2003/05/30. 10.1124/pr.55.2.2 . 12773631

[29] Roseboom PH, Klein DC. Norepinephrine stimulation of pineal cyclic AMP response element-binding protein phosphorylation: primary role of a beta-adrenergic receptor/cyclic AMP mechanism. Molecular pharmacology. 1995;47(3):439–49. Epub 1995/03/01. . 7700241

[30] Maronde E, Schomerus C, Stehle JH, Korf HW. Control of CREB phosphorylation and its role for induction of melatonin synthesis in rat pinealocytes. Biology of the cell / under the auspices of the European Cell Biology Organization. 1997;89(8):505–11. Epub 1998/06/10. . 9618900 10.1016/s0248-4900(98)80006-3

[31] Tamotsu S, Schomerus C, Stehle JH, Roseboom PH, Korf HW. Norepinephrine-induced phosphorylation of the transcription factor CREB in isolated rat pinealocytes: an immunocytochemical study. Cell and tissue research. 1995;282(2):219–26. Epub 1995/11/01. . 8565052 10.1007/BF00319113

[32] Parfitt AG, Klein DC. Sympathetic nerve endings in the pineal gland protect against acute stress-induced increase in N-acetyltransferase (EC 2.3.1.5.) activity. Endocrinology. 1976;99(3):840–51. Epub 1976/09/01. 10.1210/endo-99-3-840 . 954673

[33] Munoz EM, Bailey MJ, Rath MF, Shi Q, Morin F, Coon SL, et al NeuroD1: developmental expression and regulated genes in the rodent pineal gland. Journal of neurochemistry. 2007;102(3):887–99. Epub 2007/07/17. 10.1111/j.1471-4159.2007.04605.x . 17630985

[34] Fukuhara C, Tosini G. Analysis of daily and circadian gene expression in the rat pineal gland. Neuroscience research. 2008;60(2):192–8. 10.1016/j.neures.2007.10.011 18067983 PMC2266823

[35] Anders S, Reyes A, Huber W. Detecting differential usage of exons from RNA-seq data. Genome research. 2012;22(10):2008–17. 10.1101/gr.133744.111 22722343 PMC3460195

[36] Coon SL, Munson PJ, Cherukuri PF, Sugden D, Rath MF, Moller M, et al Circadian changes in long noncoding RNAs in the pineal gland. Proceedings of the National Academy of Sciences of the United States of America. 2012;109(33):13319–24. 10.1073/pnas.1207748109 22864914 PMC3421215

[37] Nicolae M, Mangul S, Mandoiu II, Zelikovsky A. Estimation of alternative splicing isoform frequencies from RNA-Seq data. Algorithms for molecular biology: AMB. 2011;6(1):9 10.1186/1748-7188-6-9 21504602 PMC3107792

[38] Wang Z, Gerstein M, Snyder M. RNA-Seq: a revolutionary tool for transcriptomics. Nature reviews Genetics. 2009;10(1):57–63. 10.1038/nrg2484 19015660 PMC2949280

[39] Moller M, Baeres FM. The anatomy and innervation of the mammalian pineal gland. Cell and tissue research. 2002;309(1):139–50. Epub 2002/07/12. 10.1007/s00441-002-0580-5 . 12111544

[40] Humphries A, Weller J, Klein D, Baler R, Carter DA. NGFI-B (Nurr77/Nr4a1) orphan nuclear receptor in rat pinealocytes: circadian expression involves an adrenergic-cyclic AMP mechanism. Journal of neurochemistry. 2004;91(4):946–55. 10.1111/j.1471-4159.2004.02777.x . 15525348

[41] Kim JS, Bailey MJ, Weller JL, Sugden D, Rath MF, Moller M, et al Thyroid hormone and adrenergic signaling interact to control pineal expression of the dopamine receptor D4 gene (Drd4). Molecular and cellular endocrinology. 2010;314(1):128–35. 10.1016/j.mce.2009.05.013 19482058 PMC2783391

[42] Hernandez SC, Vicini S, Xiao Y, Davila-Garcia MI, Yasuda RP, Wolfe BB, et al The nicotinic receptor in the rat pineal gland is an alpha3beta4 subtype. Molecular pharmacology. 2004;66(4):978–87. 10.1124/mol.104.002345 . 15247319

[43] Vacas MI, Sarmiento MI, Pereyra EN, Etchegoyen GS, Cardinali DP. In vitro effect of neuropeptide Y on melatonin and norepinephrine release in rat pineal gland. Cellular and molecular neurobiology. 1987;7(3):309–15. . 3440284 10.1007/BF00711307 PMC11567382

[44] Mikkelsen JD, Hauser F, Olcese J. Neuropeptide Y (NPY) and NPY receptors in the rat pineal gland. Advances in experimental medicine and biology. 1999;460:95–107. . 10810506 10.1007/0-306-46814-x_13

[45] Sugden LA, Sugden D, Klein DC. Alpha 1-adrenoceptor activation elevates cytosolic calcium in rat pinealocytes by increasing net influx. J Biol Chem. 1987;262(2):741–5. Epub 1987/01/15. . 3027065

[46] Berg GR, Klein DC. Norepinephrine increases the (32P)labelling of a specific phospholipid frac tion of post-synaptic pineal membranes. Journal of neurochemistry. 1972;19(11):2519–32. . 4263895 10.1111/j.1471-4159.1972.tb01311.x

[47] Zatz M. Denervation supersensitivity of the rat pineal to norepinephrine-stimulated [3H]inositide turnover revealed by lithium and a convenient procedure. Journal of neurochemistry. 1985;45(1):95–100. . 3998735 10.1111/j.1471-4159.1985.tb05479.x

[48] DeMeo DL, Silverman EK. Alpha1-antitrypsin deficiency. 2: genetic aspects of alpha(1)-antitrypsin deficiency: phenotypes and genetic modifiers of emphysema risk. Thorax. 2004;59(3):259–64. Epub 2004/02/27. 14985567 10.1136/thx.2003.006502 PMC1746953

[49] Gettins PG. Serpin structure, mechanism, and function. Chemical reviews. 2002;102(12):4751–804. Epub 2002/12/12. . 12475206 10.1021/cr010170+

[50] Miranda E, Lomas DA. Neuroserpin: a serpin to think about. Cellular and molecular life sciences: CMLS. 2006;63(6):709–22. 10.1007/s00018-005-5077-4 . 16465451 PMC11136441

[51] Parfitt A, Weller JL, Klein DC. Beta adrenergic-blockers decrease adrenergically stimulated N-acetyltransferase activity in pineal glands in organ culture. Neuropharmacology. 1976;15(6):353–8. Epub 1976/06/01. . 6923 10.1016/0028-3908(76)90083-6

[52] Dobin A, Davis CA, Schlesinger F, Drenkow J, Zaleski C, Jha S, et al STAR: ultrafast universal RNA-seq aligner. Bioinformatics. 2013;29(1):15–21. 10.1093/bioinformatics/bts635 23104886 PMC3530905

[53] Flicek P, Ahmed I, Amode MR, Barrell D, Beal K, Brent S, et al Ensembl 2013. Nucleic acids research. 2013;41(Database issue):D48–55. 10.1093/nar/gks1236 23203987 PMC3531136

[54] Hartley SW, Mullikin JC. QoRTs: a comprehensive toolset for quality control and data processing of RNA-Seq experiments. BMC bioinformatics. 2015;16:224 10.1186/s12859-015-0670-5 26187896 PMC4506620

[55] Anders S, Pyl PT, Huber W. HTSeq—a Python framework to work with high-throughput sequencing data. Bioinformatics. 2015;31(2):166–9. 10.1093/bioinformatics/btu638 25260700 PMC4287950

[56] Anders S, Huber W. Differential expression analysis for sequence count data. Genome Biology. 2010;11(10). 10.1186/gb-2010-11-10-r106 PMC3218662 20979621

