# Supplementary material for: Neurotranscriptomics: The Effects of Neonatal Stimulus Deprivation on the Rat Pineal Transcriptome
Source: PLoS One. 2015 Sep 14;10(9):e0137548. doi: 10.1371/journal.pone.0137548 (PMC4569390; doi:10.1371/journal.pone.0137548)
Supplement: S1 Appendix — (DOCX) [file pone.0137548.s001.docx]

Appendix

# Supplemental Data

## Supplemental Table Captions

Table S1: Genes exhibiting differential expression in both *in vivo* and *in vitro* experiments. The genes listed exhibit statistically significant (adjusted-p < 0.001) differentials in all of the following four comparisons: Control night vs day, Sham night vs day, untreated vs NE-treated, and untreated vs DBcAMP-treated.

Table S2: Genes with relatively high expression in the pineal gland. Gene symbols (when available) of all genes with > 32-fold enrichment in the pineal gland (day and/or night) and mixed non-pineal tissues. Genes that exhibit high relative expression in both the day and night analyses are listed in bold (based on the minimum of the two fold changes). A more complete list is available in the SI (Dataset S4). (docx file)

## Supplemental Datasets

Dataset S1: Complete results tables for genes differentially expressed in the pineal gland on a night/day basis, *in vivo*. (xlsx file)

Dataset S2: Complete results tables for the SCGX/DCN vs Sham analyses. (xlsx file)

Dataset S3: Complete results tables for genes differentially expressed in pineal gland in response to *in vitro* treatment with norepinephrine or dibutyryl cyclic AMP. (xlsx file)

Dataset S4: Complete results tables for mixed-tissue vs pineal-gland comparisons. (xlsx file)

## Supplemental Figures

Figure S1: Comparison of gene lists detected by the Control day-vs-night RNA-Seq analysis and a previous microarray-based analysis. Gene lists were compiled using an adjusted-p-value threshold of 0.05 and a fold change threshold of 2 (in either direction). *Note: 22 gene names that were found in the microarray results could not be matched with any current or former gene name of any known gene name in the Ensembl (release 69) or RGD (version 6) databases. Additionally: 155 microarray probe loci met the significance/fold-change criteria, but were not annotated with any known gene. These genes/probes were not counted in the figure above.

Figure S2: Summary plots for the cross-group analyses, in which SCGX and DCN groups were compared with the Sham group at both day and night. (a-d) MA plots for all four comparisons. Statistically significant genes (adjusted-p < 0.001) are marked with dots, colored based on their night/day differential status from the Control night/day analysis: blue indicates that the gene is upregulated at night, red indicates that it is downregulated at night, black indicates that the gene is not significantly differentially expressed between night and day (adjusted-p > 0.05). The displayed counts indicate the number of dots of each color above or below the FC > 2 or FC < 0.5 thresholds. A complete list of all genes with fold changes, p-values, and normalized expression estimates is available in the SI (Dataset S3). (e) Plot of the first two principal components from a principal component analysis across all 24 *in vivo* samples. Note that based on the first principal component, the Control-Night and Sham-Night samples cluster strongly to the right, while all other samples (including the SCGX and DCN samples for both day and night) cluster strongly to the left.

Figure S3: MA plot of the comparisons between mixed tissue with pineal glands at both day and night. Genes were marked in red if the mean normalized read-pair count exceeded 30 and if the (maximum-likelihood) fold change was greater than 8. Note that due to the lack of replicates no significance p-values could be calculated.

# NISC Comparative Sequencing Program Authors

Betty Barnabas, PhD; Robert Blakesley, PhD; Gerry Bouffard, PhD; Shelise Brooks, BS; Holly Coleman, MSc; Mila Dekhtyar, MSc; Michael Gregory, MSc; Xiaobin Guan, PhD; Jyoti Gupta, MSc; Joel Han, BS; Shi-ling Ho, BS; Richelle Legaspi, MSc; Quino Maduro, BS; Cathy Masiello, MSc; Baishali Maskeri, PhD; Jenny McDowell, PhD; Casandra Montemayor, MSc; James Mullikin, PhD; Morgan Park, PhD; Nancy Riebow, BS; Karen Schandler, MSc; Brian Schmidt, BS; Christina Sison, BS; Mal Stantripop, BS; James Thomas, PhD; Pam Thomas, PhD; Meg Vemulapalli, MSc; Alice Young, BA.

# Supplemental Methods

## Surgical Procedures

Five-day-old rat pups were anesthetized (Isoflurane), placed supine under a surgical microscope and the ventral neck region was disinfected with povidone-iodine. The salivary glands were exposed through a 1-cm vertical incision and retracted laterally to expose the underlying muscles. The carotid bifurcations were identified through the carotid triangle and the SCG were dissected bluntly. Further steps consisted of: a) SCGX group: SCG were removed after sectioning successively all its nervous branches with micro-dissecting spring scissors; b) DCN group: the cervical sympathetic trunks were dissected bluntly and severed 0.5 mm before entering the SCG and at 4 to 5mm caudally, removing 4 to 5 mm of the cervical sympathetic chain; c) Sham group: no further steps. Following completion of the surgical procedures on both sides of the neck the wound was cleansed and then closed with VetBond adhesive. Survival rate was 100%.

## RNA extraction and preparation

Total RNA was extracted with TRIzol reagent (Invitrogen, Carlsbad, CA), followed by clean-up using an RNeasy Micro Kit with on-column DNase treatment as per the manufacture’s protocol (Qiagen, Valencia, CA). The amount and quality of RNA were determined using a NanoDrop spectrophotometer (NanoDrop, Wilmington, DE) and an Agilent 2100 Bioanalyzer (Agilent Technologies, Santa Clara, CA). Each pool yielded 2.1 to 3.7 micrograms of total RNA (RIN values > 9).

## Library preparation and sequencing

Stranded RNA-Seq libraries were constructed from 0.7-1 µg total RNA using the TruSeq Stranded Total RNA Sample Prep Kits (Illumina cat. no. RS-122-2301) according to manufacturer’s instructions. The library insert sizes were approximately 175bp. Unique barcode adapters were applied to each library. Equal volumes of individual libraries were pool and run on a MiSeq (Illumina, San Diego, CA). The libraries were then repooled based on the MiSeq demultiplexing results. The pooled libraries were sequenced on a HiSeq2000 (Illumina, San Diego, CA) using version 3 chemistry. The data was processed using RTA version 1.17.21.3 and CASAVA 1.8.2.

The individual-biological-replicate libraries for the 24 *in vivo* samples were barcoded and combined into a single combined library. In order to yield (approximately) 40 million read-pairs per sample, this combined library was sequenced on six lanes of Illumina HiSeq 2000 sequencers. The 9 individual-sample libraries for the *in vitro* samples were also barcoded and combined into a separate combined library and sequenced on two lanes. The three samples used for the pineal marker gene experiment were indexed and pooled and run on a single lane.

This yielded an average of 48.6 million read-pairs for each sample for the *in vivo* differential expression samples (ranging from 42.9 million to 56.4 million), an average of 43.0 million read-pairs per sample for the *in vitro* differential expression samples (ranging from 39.8 million to 49.7 million), and an average of 62.8 million read-pairs per sample for the pineal marker gene samples (ranging from 55.6 million to 69.1 million).

## Alignment and quality control

The RNA-Seq data was aligned with the RNA-STAR aligner, using the rn4/RGSC3.4 genome assembly and Ensembl transcript annotation, release 69 (1, 2). Quality control metrics were calculated and visualized using the QoRTs software package, and no major artifacts or abnormalities were found. The mean alignment rate was 87.1%, with 81.4% aligning to a single unique locus. The average median insert size across all samples was 135.5, and the average mean GC content was 44.6%. Of the aligned read-pairs, 42.1% mapped at least partially to the exon of a known gene, and 0.11% mapped to the exons of more than one known gene. Of the remaining aligned read-pairs: 32.3% mapped to intronic regions of known genes, 4.0% mapped to loci within 1kb of a known gene, and 7.1% mapped to loci within 10kb of a known gene. Of the reads that mapped to known exons of annotated genes, 98.3% mapped to the correct strand. The large proportion of reads mapping to intronic regions is typical of sequencing results derived from libraries that were ribo-depleted, as ribo-depletion does not specifically select for mature mRNA (3, 4).

## Differential expression analysis

Gene read counts were provided by QoRTs, using the same algorithm described by the HTSeq documentation (using the “union” rule). For validation purposes counts were also generated using HTSeq, and we confirmed that the two methods returned identical results.

Differential expression analysis was performed using DESeq2 (5). In each analysis, genes with a mean normalized read-pair count of less than 5 were not tested. Fold change estimates were calculated via DESeq2 using the parameter estimates from the model fit. All p-values were adjusted for multiple testing using the FDR method of Benjamini, Hochberg, and Yekutieli (6, 7). Outlier detection was performed using Cook’s distance, a measure of the difference between fitted coefficients with and without each individual sample (8).

The pineal-vs-mixed-tissue experiment followed a slightly different methodology. Two separate comparisons were run: pineal-day vs mixed-tissue and pineal-night vs mixed-tissue. These comparisons were run without biological replicates, and thus the dispersion could not be rigorously estimated and no statistical hypothesis tests could be performed. Instead, the maximum likelihood fold changes were calculated between the pineal and mixed tissue samples using DESeq2. Gene lists were generated using simple thresholds on the mean read-pair count and the maximum likelihood fold changes between the pineal and mixed-tissue samples. We selected a normalized read-pair count threshold of 30, and a fold change threshold of 8.

## Comparison with previous microarray analyses

The results of the differential expression analyses found in the *in vivo* differential experiments were compared with a previous microarray-based study (9). This study detected 517 loci with significant differential expression using an adjusted p-value threshold of 0.05 and a fold-change threshold of 2. Of these 517 loci, 155 were not linked with any known gene, and 22 of the loci were labelled with gene identifiers that do not appear to be a current or former identifier for any known gene in either the Ensembl (release 69) or RGD (version 6) databases. The results of the *in vivo* experiments were compared with those of this previous study, using the same cutoff thresholds in each (adjusted-p < 0.05, fold change > 2 or < 0.5).

# References

1. Dobin A*, et al.* (2013) STAR: ultrafast universal RNA-seq aligner. *Bioinformatics* 29(1):15-21.

2. Flicek P*, et al.* (2013) Ensembl 2013. *Nucleic acids research* 41(Database issue):D48-55.

3. Adiconis X*, et al.* (2013) Comparative analysis of RNA sequencing methods for degraded or low-input samples. *Nature methods* 10(7):623-629.

4. Benes V, Blake J, & Doyle K (2011) Ribo-Zero Gold Kit: improved RNA-seq results after removal of cytoplasmic and mitochondrial ribosomal RNA. *Nature methods* 8(11).

5. Anders S & Huber W (2010) Differential expression analysis for sequence count data. *Genome Biology* 11(10).

6. Benjamini Y & Hochberg Y (1995) Controlling the false discovery rate: a practical and powerful approach to multiple testing. *Journal of the Royal Statistical Society Series B* 57:289-300.

7. Benjamini Y & Yekutieli D (2001) The control of the false discovery rate in multiple testing under dependency. *Annals of Statistics* 29:1165-1188.

8. Cook RD (1977) Detection of influential observation in linear-regression. *Technometrics* 19(1):15-18.

9. Bailey MJ*, et al.* (2009) Night/day changes in pineal expression of >600 genes: central role of adrenergic/cAMP signaling. *J Biol Chem* 284(12):7606-7622.
