# Supplementary figures and images for: Neurotranscriptomics: The Effects of Neonatal Stimulus Deprivation on the Rat Pineal Transcriptome
Source: PLoS One. 2015 Sep 14;10(9):e0137548. doi: 10.1371/journal.pone.0137548 (PMC4569390; doi:10.1371/journal.pone.0137548)

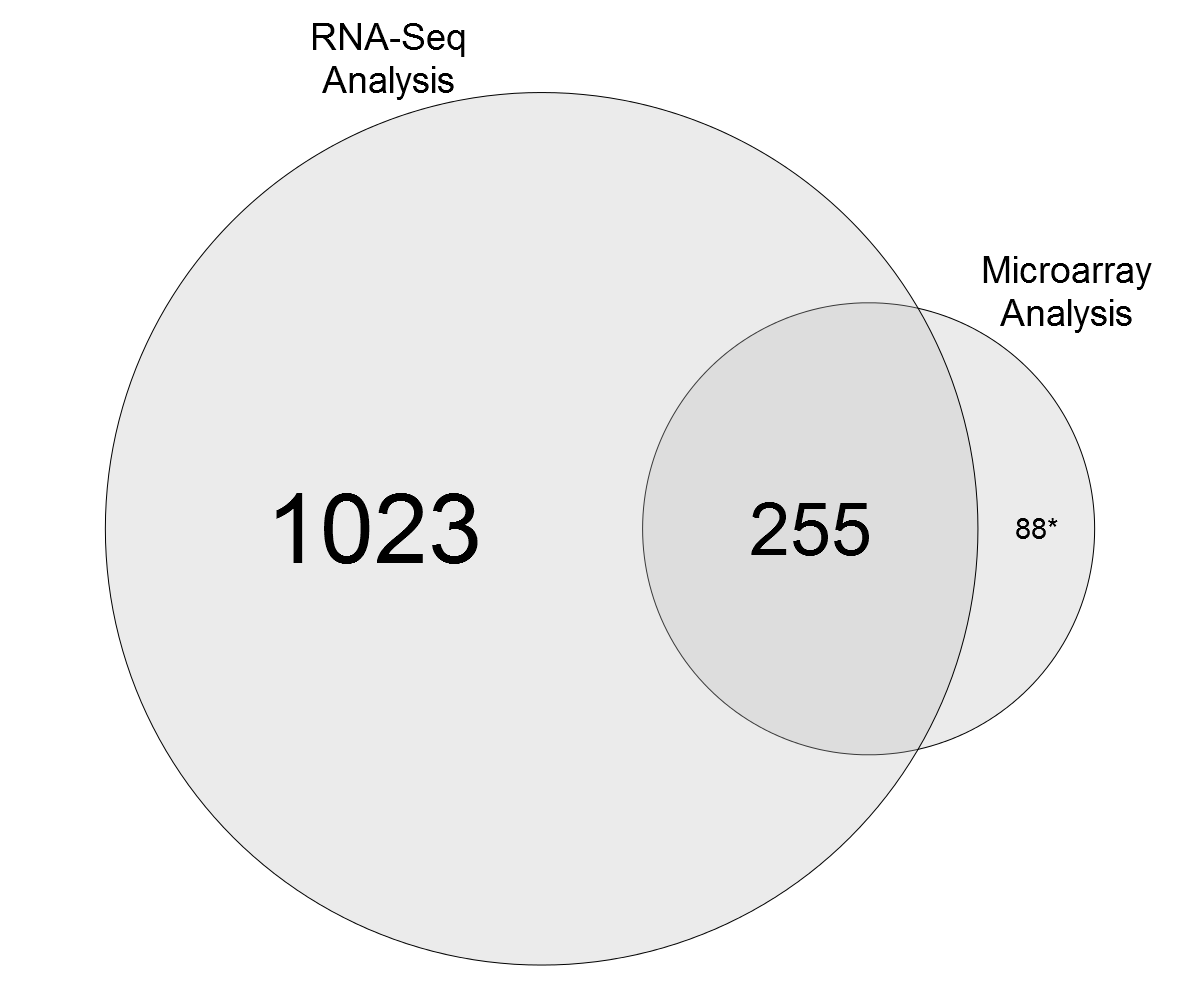

Supplement: S1 Fig — Gene lists were compiled using an adjusted-p-value threshold of 0.05 and a fold change threshold of 2 (in either direction). *Note: 22 gene names that were found in the microarray results could not be matched with any current or former gene name of any known gene name in the Ensembl (release 69) or RGD (version 6) databases. Additionally: 155 microarray probe loci met the significance/fold-change criteria, but were not annotated with any known gene. These genes/probes were not counted in the figure above. (TIF) [file pone.0137548.s006.tif]

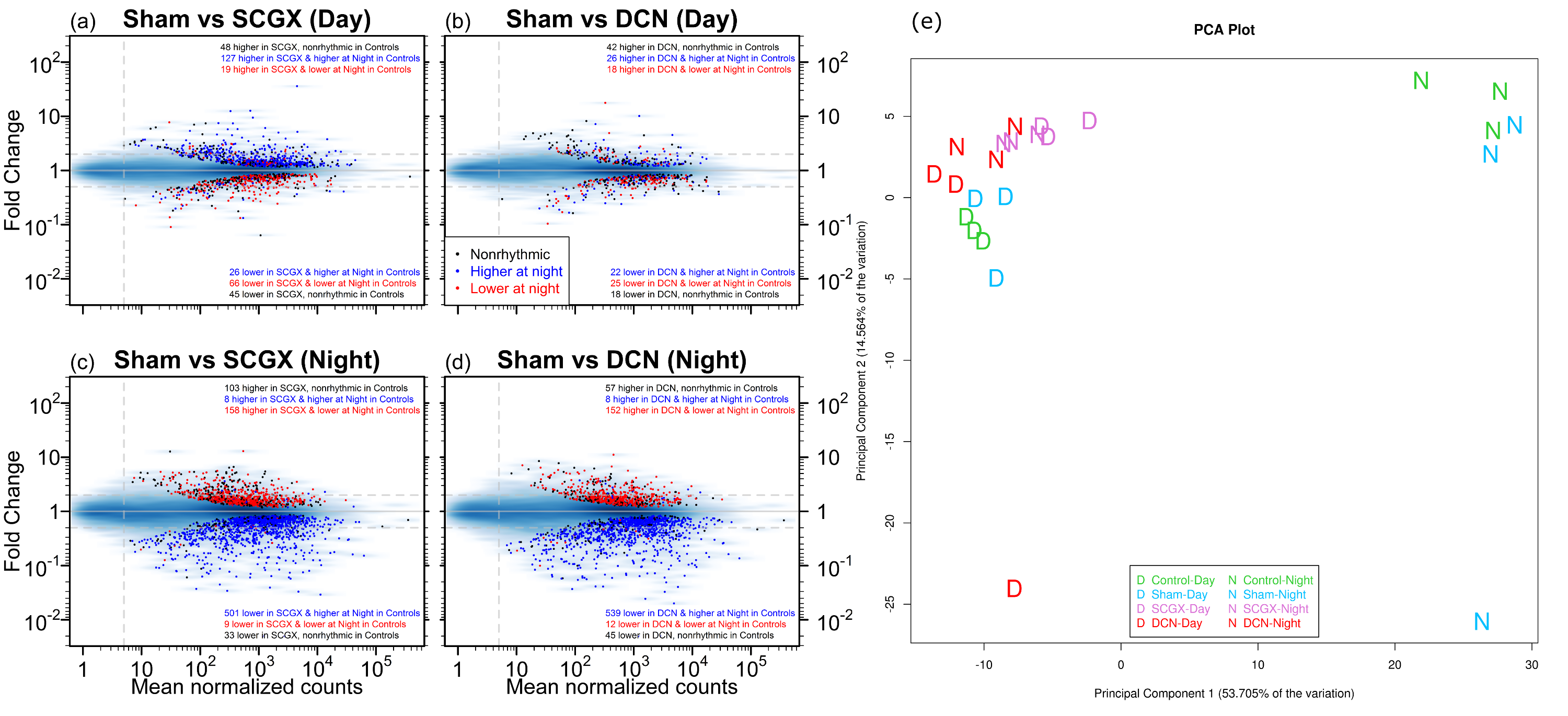

Supplement: S2 Fig — (a-d) MA plots for all four comparisons. Statistically significant genes (adjusted-p < 0.001) are marked with dots, colored based on their night/day differential status from the Control night/day analysis: blue indicates that the gene is upregulated at night, red indicates that it is downregulated at night, black indicates that the gene is not significantly differentially expressed between night and day (adjusted-p > 0.05). The displayed counts indicate the number of dots of each color above or below the FC > 2 or FC < 0.5 thresholds. A complete list of all genes with fold changes, p-values, and normalized expression estimates is available in the SI (S3 Dataset). (e) Plot of the first two principal components from a principal component analysis across all 24 in vivo samples. Note that based on the first principal component, the Control-Night and Sham-Night samples cluster strongly to the right, while all other samples (including the SCGX and DCN samples for both day and night) cluster strongly to the left. (TIF) [file pone.0137548.s007.tif]

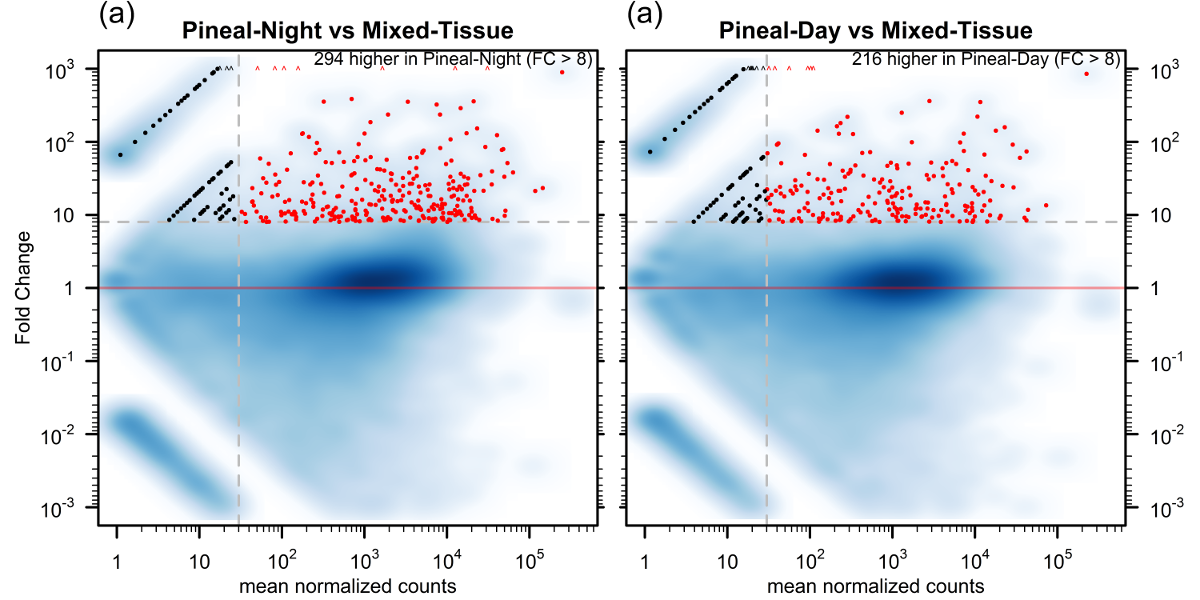

Supplement: S3 Fig — Genes were marked in red if the mean normalized read-pair count exceeded 30 and if the (maximum-likelihood) fold change was greater than 8. Note that due to the lack of replicates no significance p-values could be calculated. (TIF) [file pone.0137548.s008.tif]
