# Supplementary material for: Neurotranscriptomics: The Effects of Neonatal Stimulus Deprivation on the Rat Pineal Transcriptome
Source: PLoS One. 2015 Sep 14;10(9):e0137548. doi: 10.1371/journal.pone.0137548 (PMC4569390; doi:10.1371/journal.pone.0137548)
Supplement: S1 Table — The genes listed exhibit statistically significant (adjusted-p < 0.001) differentials in all of the following four comparisons: Control night vs day, Sham night vs day, untreated vs NE-treated, and untreated vs DBcAMP-treated. (DOCX) [file pone.0137548.s009.docx]

Table S1: Genes exhibiting differential expression in both *in vivo* and *in vitro* experiments. The genes listed exhibit statistically significant (adjusted-p < 0.001) differentials in all of the following four comparisons: Control night vs day, Sham night vs day, untreated vs NE-treated, and untreated vs DBcAMP-treated.

| **Weakest Fold Change, all 4 analyses** | **Gene Symbol** |
| --- | --- |
| **>32** | Aanat^*†^, Slc15a1^†^ |
| **16 - 32** | Dusp1^†^ |
| **8 - 16** | Cd24^†^, Chd5, Dclk3^*†^, Fcer1a^*†^, Irs2^*†^, Nr4a1^†^, Pde10a^*†^, Ptch1^*†^, Sik1^*†^ |
| **4 - 8** | Atp7b^*†^, B3gnt8^*^, Bhlha15, Camk1g^*†^, Coq10b^*†^, CREM^*†^, Dos^†^, Etnk1^*†^, F1LWE5, Fdx1^†^, Gem^*†^, Grm2, Hcrtr1^†^, Ipcef1, Irak2^*†^, Kcnq4^*^, Lamb3^†^, MGC94891^*^, Nap1l5^†^, nod3l^†^, Nptx1^*†^, Osbpl6, Padi4^*†^, Pde4b^*†^, Rem2, RGD1560523^*†^, Rgs2^†^, Slc17a6^†^, St8sia2^*^, Syt4^†^, Tbc1d1^†^, Tinagl1^†^, Wnt10a^†^, Xpot^†^, Zrsr1^*†^ |
| **1/4 - 1/8** | LOC690918^*^ |
| **<1/8** | - |

^*^Gene has high relative expression in the pineal gland; ^†^gene has previously been found to be differentially expressed between day and night conditions in microarray experiments.
