# Supplementary material for: Neurotranscriptomics: The Effects of Neonatal Stimulus Deprivation on the Rat Pineal Transcriptome
Source: PLoS One. 2015 Sep 14;10(9):e0137548. doi: 10.1371/journal.pone.0137548 (PMC4569390; doi:10.1371/journal.pone.0137548)
Supplement: S2 Table — Gene symbols (when available) of all genes with > 32-fold enrichment in the pineal gland (day and/or night) and mixed non-pineal tissues. Genes that exhibit high relative expression in both the day and night analyses are listed in bold (based on the minimum of the two fold changes). A more complete list is available in the SI (S4 Dataset). (DOCX) [file pone.0137548.s010.docx]

Table S2: Genes with relatively high expression in the pineal gland. Gene symbols (when available) of all genes with > 32-fold enrichment in the pineal gland (day and/or night) and mixed non-pineal tissues. Genes that exhibit high relative expression in both the day and night analyses are listed in bold (based on the minimum of the two fold changes). A more complete list is available in the SI (Dataset S4).

| **Fold Change** | **Gene Symbol** |
| --- | --- |
| **>128** | Aanat^‡§^, **Adam2**, **Asmt**, **Bsx**^‡§^, **Calhm1**^‡^, **Chrnb4**, **Col8a1**, D3ZW53^‡^, **Defb24**, F1M032, **F1MAE8,** Fcer1a^‡§^, **Isl2**, Krt15, **Lhx4**, **LOC100362601**, LOC679835, Neurog1^‡^, Padi4^‡§^, **Pde6c**, **RGD1563903**, **Rhox12**, Ribc2^‡§^, Rptn^‡^, **Tph1**, Tpo |
| **32 - 128** | Abca17, Abcc12, Adrb1, **Alox15**, **Alx4**, Arhgap24, Atp7b^‡§^, B3gnt8^‡§^, Cabp5, **Chrna3**, **Cngb3**, Cpg1, Cplx3, **D4A4K8,** D4A976, Dclk3^‡§^, EMAL5^‡^, **Esm1**, **F1LTW7,** F1M5V3, F1M848^‡^, F1MAG9, Fam83a, Fyb, **Gch1**, Gdf15^‡^, Gem^‡§^, **Gipc3**, **Gnat2**, **Gnb3**, **Gngt2**, Hpse2, Hs3st2^‡^, IGG2A, Irs1, **Kcnh6**, Kcnq4^‡§^, **Krt13**, **Krt15**, LOC100360666, LOC500567, LOC500990, LOC691317^‡^, **Lrrc38**, **Mitf**, Mpp4, **Nek11**, **Nphs1**, Nyx, Ocm, Opn1sw^‡^, **Pax3**, Prss33, **Prtg**, Q5XHY2, RGD1309108^‡^, RGD1559884^‡^, RGD1560523^‡§^, RGD1561530, **RGD1562024**, Rnase1l2^‡^, Rnf222, Sh2d4b, Sik1^‡§^, Slc4a5, Tbata, Tex14, Tmprss9, **Ttc23l**, Wfikkn2^‡^ |

^‡^Gene has strong (FC > 4 or FC < 0.25) and statistically significant (adjusted-p < 0.001) differential expression in the Control and/or Sham day/night *in vivo* analyses.

^§^Gene has strong and statistically significant differential expression in either or both of the treated vs untreated *in vitro* analyses.
